# Conflicting relationship between age-dependent disorders, valvular heart disease and coronary artery disease by covariance structure analysis: Possible contribution of natriuretic peptide

**DOI:** 10.1371/journal.pone.0181206

**Published:** 2017-07-20

**Authors:** Risa Fukumoto, Makoto Kawai, Kosuke Minai, Kazuo Ogawa, Jun Yoshida, Yasunori Inoue, Satoshi Morimoto, Toshikazu Tanaka, Tomohisa Nagoshi, Takayuki Ogawa, Michihiro Yoshimura

**Affiliations:** Division of Cardiology, Department of Internal Medicine, The Jikei University School of Medicine, Tokyo, Japan; Scuola Superiore Sant'Anna, ITALY

## Abstract

**Background:**

It is conceivable that contemporary valvular heart disease (VHD) is affected largely by an age-dependent atherosclerotic process, which is similar to that observed in coronary artery disease (CAD). However, a comorbid condition of VHD and CAD has not been precisely examined. The first objective of this study was to examine a possible comorbid condition. Provided that there is no comorbidity, the second objective was to search for the possible reasons by using conventional risk factors and plasma B-type natriuretic peptide (BNP) because BNP has a potentiality to suppress atherosclerotic development.

**Methods:**

The study population consisted of 3,457 patients consecutively admitted to our institution. The possible comorbid condition of VHD and CAD and the factors that influence the comorbidity were examined by covariance structure analysis and multivariate analysis.

**Results:**

The distribution of the patients with VHD and those with CAD in the histograms showed that the incidence of VHD and the severity of CAD rose with seniority in appearance. The real statistical analysis was planned by covariance structure analysis. The current path model revealed that aging was associated with VHD and CAD severity (*P* < 0.001 for each); however, as a notable result, there was an inverse association regarding the comorbid condition between VHD and CAD (Correlation coefficient [β]: -0.121, *P* < 0.001). As the second objective, to clarify the factors leading to this inverse association, the contribution of conventional risk factors, such as age, gender, hypertension, smoking, diabetes, obesity and dyslipidemia, to VHD and CAD were examined by multivariate analysis. However, these factors did not exert an opposing effect on VHD and CAD, and the inverse association defied explanation. Since different pathological mechanisms may contribute to the formation of VHD and CAD, a differentially proposed path model using plasma BNP revealed that an increase in plasma BNP being drawn by VHD suppressed the progression of CAD (β: -0.465, *P* < 0.001).

**Conclusions:**

The incidence of VHD and CAD showed a significant conflicting relationship. This result supported the likely presence of unknown diverse mechanisms on top of the common cascade of atherosclerosis. Among them, the continuous elevation of plasma BNP due to VHD might be one of the explicable factors suppressing the progression of CAD.

## Introduction

### Evolving environments surrounding valvular heart disease

Rheumatic fever was previously thought to be the common cause of valvular heart disease (VHD). A decreased incidence of rheumatic fever has been attributed to the introduction of penicillin or a change in the virulence of *Streptococcu*s, as well as improved social conditions [1]. In this manner, contemporary VHD would have been largely altered by lifestyle change, environment, medical development, and aging of the population. Regarding current VHD, calcific aortic valve disease is a slowly progressive disorder with a disease continuum that ranges from mild valve thickening to severe calcification with impaired leaflet motion. Compelling histopathologic and clinical data suggest that calcific aortic valve disease is an active disease process akin to atherosclerosis with lipoprotein deposition and chronic inflammation [2]. On another front, mitral annular calcification is a chronic degenerative process of the mitral valve ring. Calcification of the annulus fibrosis of the mitral valve was commonly found in elderly people at autopsy and was postulated to be a sequela of rheumatic heart disease [3, 4]. Howeve, evidence of previous disease is often absent, and the lesion is currently regarded as resulting from atherosclerosis and calcification. Mitral annular calcification and atherosclerosis share similar risk factors, and the presence of mitral annual disruption may reflect the intensity and duration of exposure to these risk factors [5, 6]. Interestingly, mitral annual calcification has been proposed as a visible barometer of the degree of atherosclerosis [7, 8].

### An undefined question regarding a possible comorbid condition of VHD and coronary artery disease in the modern period

The coronary artery is a region with a predilection for atherosclerosis. In addition, calcification is highly prevalent in the coronary artery of patients with coronary artery disease (CAD). The overlap in the clinical risk factors associated with VHD and CAD suggests a shared disease process. It is thus natural that VHD and CAD should advance simultaneously with seniority and seemingly coexist in elderly patients. Among the risk factors, aging is rightfully a common as well as a key factor advancing heart disorders involving atherosclerosis and calcification in both VHD and CAD [9]. However, we consider the comorbid condition between VHD and CAD to be still unclear.

### Possible contribution of natriuretic peptide as an intervening factor between VHD and CAD

From an experience perspective, VHD and CAD may not always exist simultaneously in the real world. Although VHD and CAD development are dependent on the degree of atherosclerosis and calcification in the same fashion, the presence of an intervention factor that suppresses one or the other should to be considered. It is interesting to search a possible factor among conventional risk factors. Furthermore, another possible interjacent factor may be activation of natriuretic peptide. A-type and B-type natriuretic peptides (ANP and BNP) are activated in the atria and ventricles, respectively [10–12], as well as in many cardiovascular disorders and conditions [13–21]. In patients with VHD, cardiomyocytes are likely stretched due to valvular abnormalities with a diverse range of influences. Particularly, left ventricular volume overload due to aortic valve regurgitation (AR) and mitral valve regurgitation (MR) might just as well activate BNP. Natriuretic peptides are physiologically active substances that are involved in vasodilation, natriuretic effects, and the inhibition of the renin–angiotensin–aldosterone system, and other functions. The second messenger of natriuretic peptides is cyclic guanosine monophosphate (cGMP). Intriguingly, the cGMP cascade was reported to be beneficial for suppression of the atherosclerosis process and the anti-hypertrophic effects on the heart [22, 23].

### Objective of this study

The first objective of this study was to statistically examine the possible comorbid condition of VHD and CAD. In this analysis, we used covariance structure analysis because the path model could be constructed along with the histograms and distribution of the study patients with and without VHD and the severity of CAD. If there was no comorbidity between VHD and CAD in the first study, the second study was projected. Its objective was to search for a possible intervenient factors. We examined conventional risk factors; in particular, we focused on the possibility of plasma BNP. To this end, we again used covariance structure analysis because possible causality could be examined by this method.

## Methods

### Study patients

The study patients were consecutively admitted to our institution for any cause and underwent both echocardiography and cardiac catheterization. Clinically meaningful VHD, aortic valve stenosis (AS), AR, mitral valve stenosis (MS), and MR were examined, and the degree of organic stenosis in the coronary arteries was evaluated precisely by coronary angiography. The ethics committee of The Jikei University School of Medicine approved the study protocol (24–355[7121]), and we complied with our institution’s routine ethical regulations. Informed consent was obtained from each patient, and all clinical investigations were conducted in accordance with the principles expressed in the Declaration of Helsinki. According to our routine ethical regulations, we posted a notice about the study design and contact information at a public location in our institution.

### Definition of diseases

AS was defined as an aortic valve area of less than 1.5 cm^2^ plus either a mean valve gradient of at least 25 mmHg or a peak velocity of at least 3.0 m/sec [24]. AR and MR were diagnosed by the multiple two-dimensional imaging parameters, as described in the American Society of Echocardiography guidelines [25]. AR was defined as a jet deceleration rate (pressure half time) of less than 500 ms and/or a vena contracta width of more than 0.3 cm. MR was defined as a color flow jet area of more than 20% of left atrial area, a vena contracta width of more than 0.3 cm, a regurgitant fraction of more than 30%, and/or an effective regurgitant orifice area of at least 0.20 cm^2^. Finally, MS was defined as a mitral valve area of less than 1.5 cm^2^ plus a mean valve gradient of at least 5 mm Hg [24].

CAD was diagnosed by cardiac catheterization, and the severity of CAD was determined by the number of the arteries with organic stenosis. Significant organic stenosis was defined as 75% or more stenosis in the coronary arteries by coronary angiography. In this study, we simply divided the patients into four groups (0-, 1-, 2-, and 3-vessel disease [VD] groups) according to the number of diseased vessels with significant organic stenosis because the morphologic alteration due to atherosclerosis and calcification in the coronary arteries was compared with that in valves in this study. Patients with acute coronary syndrome, coronary spastic angina, and chest pain syndrome were included in the study population.

As baseline diseases, hypertension, diabetes mellitus, and dyslipidemia were defined as previously described [26]. Patients with renal dysfunction were defined as those having an estimated glomerular filtration rate (eGFR) of <60 mL/min/1.73 m^2^ at admission according to the Japanese Society of Nephrology guidelines. We calculated the eGFR according to the Modification of Diet in Renal Disease Study equation [27] shown below, with coefficients modified for Japanese patients [28].

$$\text{eGFR }\left(\text{mL}/\text{min}/1.73{\text{ m}}^{2}\right)\;=\;194\;\times {\text{ age}}^{-0.287}\times {\text{ Creatinine}}^{-1.094}\left(\text{and }\times \;0.739\text{ for females}\right).$$

### Blood sampling and hemodynamic examination during cardiac catheterization

We collected blood samples and hemodynamic data during cardiac catheterization. Routine biochemical analyses, such as electrolytes, renal function, liver function, and lipid and glucose profiles, were performed in a central laboratory in our hospital during the study. The measurement of plasma BNP levels was also simultaneously performed as described in the previous report [16–21]. The approach to BNP measurement is essential because there are differences between the analytical characteristics and clinical results among the BNP assay systems [29]. In brief, the plasma BNP level was measured in our institution by a central laboratory using the E Test TOSOH II (Tosoh Corporation, Tokyo, Japan; http://www.diagnostics.jp.tosohbioscience.com/immunoassay/aia-reagents). This assay method was fundamentally similar to the Shionoria BNP (Shionogi Co. Ltd., Tokyo, Japan). Good correlations were found between the measurements obtained with the Shionoria BNP and those obtained using the other method at the individual facilities, with correlation equations and coefficients of y = 0.97x + 3.83 (r = 0.996) with the Shionoria BNP denoted. The intra-day reproducibility (determined using the coefficient of variation [CV]) was 2.0–2.7% and the inter-day reproducibility CV was 1.4–3.2%.

The left ventricular ejection fraction (LVEF) was calculated by left ventriculography during cardiac catheterization.

### Statistical analysis

Continuous variables are expressed as the means ± the standard deviations (SDs) or the medians with the ranges. Categorical variables are expressed as percentages. Comparisons between two continuous variables were performed using Pearson’s product-moment correlation coefficient analysis. The Kolmogorov-Smirnov test was used to determine whether the B-type natriuretic peptide (BNP) values were normally distributed. Subsequently, the BNP data were log-transformed (Log BNP) to achieve a normal distribution for the analysis. The smoking context was divided into current smokers or nonsmokers. All statistical analyses were performed using SPSS Statistics version 23.0 (SPSS Inc., Chicago, IL, USA), and differences were considered statistically significant for *P* values < 0.05.

**Fig 1:**
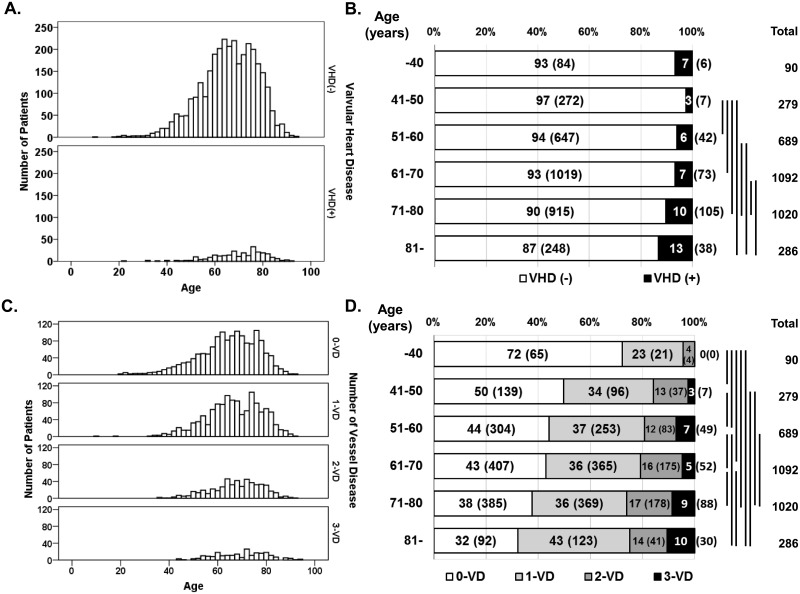
Histograms and distribution of study patients with valvular heart disease or coronary artery disease. The histogram (A) and distribution (B) of the study population with and without VHD, and the histogram (C) and distribution (D) of the study population with 0-VD, 1-VD, 2-VD, and 3-VD were diagrammed. The vertical bars denote the statistically significant differences from the Chi-square analysis for the distribution of the study population with and without VHD (*P* < 0.05 or more) and for the distribution of the study population with 0-VD, 1-VD, 2-VD, and 3-VD (*P* < 0.05 or more). No marked pair means there are no statistically significant differences from each other by Chi-square analysis. VD, vessel disease.

Covariance structure analysis was used in this study. The reason for using this analysis was that the path model could be constructed as the model akin to the fundamental image of Fig 1. In the analysis, we examine the possible comorbid condition of VHD and CAD in consideration of aging (and/or age-associated factors). In addition, this analysis was used when examining the complicated relationships among VHD, CAD and plasma BNP. We aimed to express the possible effect of plasma BNP on CAD suppression by making an allowance for the CAD-induced BNP secretin in an opposite manner. This analysis was performed with IBM SPSS AMOS version 23 (Amos Development Corporation., Meadville, PA, USA). The obtained structural equation models were tested and confirmed at the significance level for *P* values < 0.05. The causality model defines some hierarchical regression models between clinical factors and VHD or CAD. As we reported previously [19–21, 30–32], paths between variables are drawn from independent to dependent variables with a directional arrow for every regression model (arrowhead on one end only). A two-way arrow between two variables indicates the correlation between these two variables. For every regression, the total variance in dependent variable is theorized to be caused either by independent variables of the model or by extraneous variables (**e**). Each path has a coefficient showing the standardized coefficient of regressing independent variables on dependent variables of the relevant path. Although the covariance structure analysis is a powerful statistical analysis, it is well known to also be affected by several factors [33]. Still, covariance structure analysis has come into use in medical science. Indeed, we recently reported clinical studies by using covariance structure analysis [19–21 30–32].

## Results

### Characteristics of the study patients

Table 1 shows the patients’ characteristics in this study. The total study population comprised 3,457 patients. They included 271 patients with VHD (the whole group of VHDs), 132 patients with AS, 79 patients with AR, 20 patients with MS, and 100 patients with MR. Thirty-five patients had overlapping VHD disorders.

**Table 1:** Characteristics of all the patients.

Characteristics (N = 3,457)	
Number (%) or Mean ± SD [Median; Interquartile range]	
Gender; Male	2,837 (82.1)
Age (years old)	65.6 ± 11.7
BMI (kg/m^2^)	24.2 ± 4.3
Current smoker	710 (20.5)
Family history of IHD	831 (24.0)
Hb (g/dL)	13.1 ± 2.1
Creatinine (mg/dL)	1.74 ± 2.55
eGFR (mL/min/1.73 m^2^)	61.7 ± 26.9
UA (mg/dL)	6.0 ± 1.5
FBS (mg/dL)	121.1 ± 42.7
HbA1c (%)	6.2 ± 1.0
TG (mg/dL)	123.4 ± 92.2
HDL-C (mg/dL)	51.1 ± 15.4
LDL-C (mg/dL)	100.5 ± 30.8
LDL-C/HDL-C	2.12 ± 0.89
CRP (mg/dL)	0.61 ± 1.81
BNP (ng/L)	209.4 ± 559.0 [53.7; 18.9–168.3]
Left ventricular hemodynamic parameters	
LVEF (%)	56.2 ± 12.1
Underlying cardiovascular disease number	
Ischemic heart disease	1,275 (36.9)
Acute coronary syndrome	208 (6.02)
Angina pectoris	1,100 (31.8)
Coronary artery disease	
0—vessel disease	1,455 (42.1)
1—vessel disease	1,257 (36.4)
2—vessel disease	518 (15.0)
3—vessel disease	226 (6.5)
Cardiomyopathy	272 (7.9)
Valvular disease	271 (7.8)
Aortic valve stenosis	132 (3.8)
Aortic valve regurgitation	79 (2.3)
Mitral valve stenosis	20 (0.6)
Mitral valve regurgitation	100 (2.9)
Arrhythmia	375 (10.8)
Atrial fibrillation	206 (6.0)
Other than AF	169 (4.9)
Hypertension	2,632 (76.1)
Type-2 diabetes mellitus	1,420 (41.1)
Dyslipidemia	2,465 (71.3)
Hemodialysis	370 (10.7)
Medication	
Antiplatelet agent	2,457 (71.1)
Anticoagulant agent	439 (12.7)
ACE inhibitors	633 (18.3)
ARBs	1,375 (39.8)
Beta blockers	1,417 (41.0)
Calcium channel blockers	1,842 (53.3)
Diuretics	725 (21.0)
Statins	1,934 (55.9)
Non-statin for dyslipidemia	389 (11.3)
Oral antidiabetic agents	907 (26.2)
Insulin	373 (10.8)
Anti-hyperuricemia	628 (18.2)

Hb, hemoglobin; UA, uric acid; FBS, fasting blood sugar; HbA1c, hemoglobin A1c; TG, triglycerides; HDL-C, high-density lipoprotein cholesterol; LDL-C, low-density lipoprotein cholesterol; ACE, angiotensin-converting enzyme; ARBs, angiotensin II type I-receptor blockers; BMI, body mass index; BNP, B-type natriuretic peptide; CRP, C-reactive protein; eGFR, estimated glomerular filtration rate; and LVEF, left ventricular ejection fraction

### Distribution of the patients with VHD and those with CAD

Fig 1-A and 1-B show the histograms and distribution of the study patients with and without VHD. In addition, Fig 1-C and 1-D show the histograms and distribution of the study patients with 0-VD (n = 1,455), 1-VD (n = 1,257), 2-VD (n = 518), and 3-VD (n = 226). The VHD incidence and the CAD severity rose with seniority in appearance. However, the real comorbidity was unknown in these figures.

### Concept of proposed path model A using the whole group of VHDs

In the first path model A, we compared the total number of patients with VHD to those with CAD. We constructed a path model akin to the concept of Fig 1. As a matter of logic, the theoretical path model A was proposed by positioning VHD and CAD in parallel and centrally because this is the main perspective. The association between them was linked by the two-way arrows. Aging (and/or age-associated factors) was placed, following arrows to VHD and CAD. Paths between variables were drawn from independent to dependent variables with a directional arrow for every regression model, which were able to examine causality.

### Result of path model A

**Fig 2:**
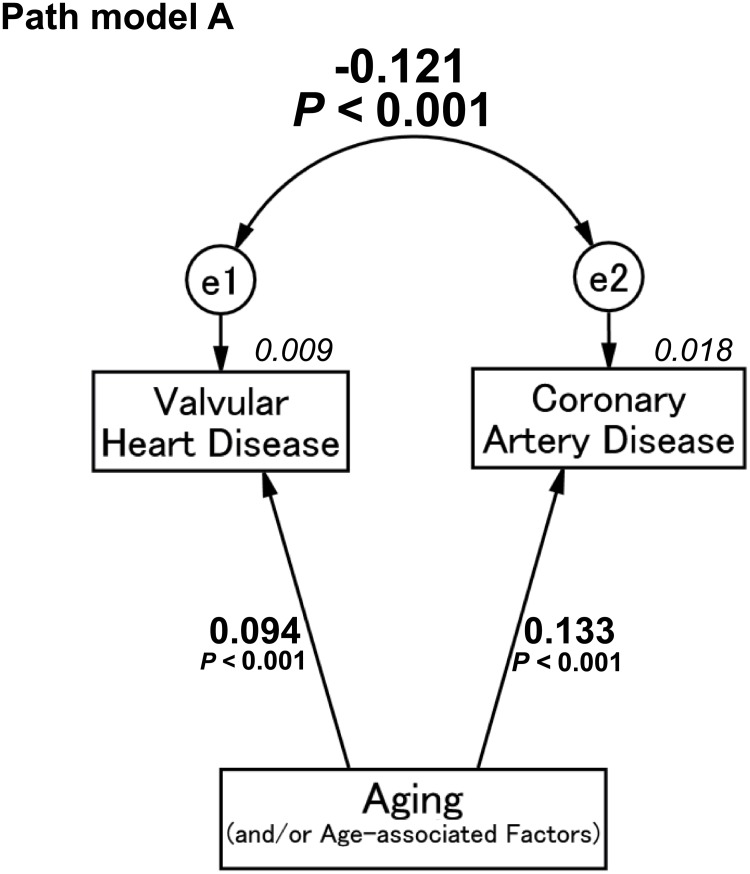
Path model (A): Association between whole valvular heart disease and coronary artery disease with an explanatory drawing of the possible cascade from aging. This path has a coefficient showing the standardized coefficient of regressing independent variables on the dependent variable of the relevant path. These variables indicate standardized regression coefficients, squared multiple correlations [italic capitals], and correlations among exogenous variables [capitals upper side of the two-way arrowhead curves].

As shown in Fig 2 and Table 2, path model A revealed that aging (and/or age-associated factors) was associated with the whole group of VHDs and the severity of CAD (*P* < 0.001, for each). However, intriguingly, we found no positive association but a negative (inverse) association between the whole group of VHDs and CADs ([e1-e2], the correlation coefficient [β]: -0.121, *P* < 0.001).

**Table 2:** Results of path model A.

Clinical factor				Regression coefficient [95% CI]	*P* value	Standardized regression coefficient [95% CI]
VHD (R^2^ = 0.009)		←	Age	0.002 [0.001, 0.003]	<0.001	0.094 [0.061, 0.127]
CAD (R^2^ = 0.018)		←	Age	0.010 [0.008, 0.013]	<0.001	0.133 [0.100, 0.165]
Correlation coefficient		*P* value				
e1 ↔ e2	-0.121	<0.001				

The direct effects of the path model theoretically proposed (see Fig 2).

CAD, Coronary artery disease; VHD, valvular heart disease; e, extraneous variable; CI, confidence interval; and R^2^, squared multiple correlations

### Concept of proposed path model B using respective subtypes of VHD

The next path model was planned to investigate a relationship between respective subtypes of VHD and CAD. As with the case of path model A, the theoretical path model B was proposed by positioning AS, AR, MS, MR, and CAD in parallel. Aging (and/or age-associated factors)was placed, following the arrows to VHD and CAD.

### Result of path model B

**Fig 3:**
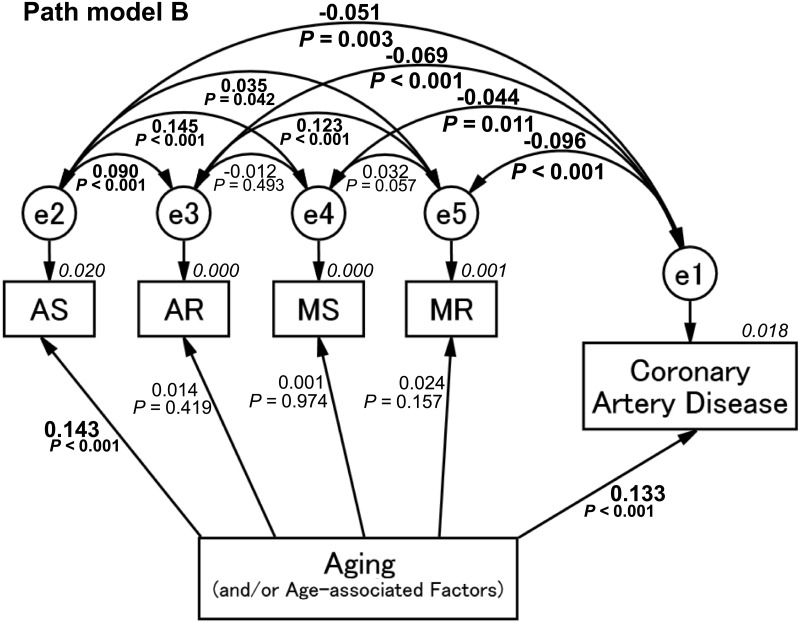
Path model (B): Association between the subtype of valvular heart disease and coronary artery disease with an explanatory drawing of the possible cascade from aging. This path has a coefficient showing the standardized coefficient of regressing independent variables on the dependent variable of the relevant path. These variables indicate standardized regression coefficients, squared multiple correlations [italic capitals], and correlations among exogenous variables [capitals upper/lower side of the two-way arrowhead curves]. AS, aortic valve stenosis; AR, aortic valve regurgitation; MS, mitral valve stenosis; MR, mitral valve regurgitation.

As shown in Fig 3 and Table 3, path model B revealed that, among the subtypes of VHD, aging (and/or age-associated factors) was significantly associated with AS (*P* < 0.001) but not with other subtypes of VHD (AR, MS, and MR: *P* = 0.419, 0.974, 0.157, respectively). Nevertheless, there was an inverse association between AS and CAD (AS: [e1-e2], β: -0.051, *P* = 0.003). Furthermore, AR, MS, and MR also showed a significant inverse association with CAD (AR: [e1-e3], β: -0.069, *P* < 0.001; MS: [e1-e4], β: -0.044, *P* = 0.011; MR: [e1-e5], β: -0.096, *P* < 0.001). These results suggest that the subtypes of VHD, including AS, did not necessarily occur together with CAD.

**Table 3:** Results of path model B.

Clinical factor			Regression coefficient [95% CI]	*P* value	Standardized regression coefficient [95% CI]			
AS (R^2^ = 0.020)	←	Age	0.002 [0.002, 0.003]	<0.001	0.143 [0.110, 0.176]			
AR (R^2^ = 0.000)	←	Age	0.000 [0.000, 0.001]	0.419	0.014 [-0.019, 0.176]			
MS (R^2^ = 0.000)	←	Age	0.000 [0.000, 0.000]	0.974	0.001 [-0.033, 0.034]			
MR (R^2^ = 0.001)	←	Age	0.000 [0.000, 0.001]	0.157	0.024 [-0.009, 0.057]			
CAD (R^2^ = 0.018)	←	Age	0.010 [0.010, 0.013]	<0.001	0.132 [0.100, 0.165]			
Correlation coefficient	Correlation coefficient	*P* value	Correlation coefficient	Correlation coefficient	*P* value	Correlation coefficient	Correlation coefficient	*P* value
e1 ↔ e2	-0.051	0.003	e2 ↔ e3	0.090	<0.001	e3 ↔ e4	-0.012	0.493
e1 ↔ e3	-0.069	<0.001	e2 ↔ e4	0.145	<0.001	e3 ↔ e5	0.123	<0.001
e1 ↔ e4	-0.044	0.011	e2 ↔ e5	0.035	0.042	e4 ↔ e5	0.032	0.057
e1 ↔ e5	-0.096	<0.001						

The direct effects of the path model theoretically proposed (see Fig 3).

CAD, Coronary artery disease; AS, Aortic valve stenosis; AR, Aortic valve regurgitation; MS, Mitral valve stenosis; MR, Mitral valve regurgitation; e, extraneous variable; CI, confidence interval; and R^2^, squared multiple correlations

### Chi-square test between the whole group of VHDs and multi-vessel CAD

In a separate analysis, the association between the whole group of VHDs and multi-vessel disease (two and three vessels with organic stenosis in the coronary artery) was examined using the Chi-square test. As shown in Table 4, there was a significant difference in the incidence (*P =* 0.031 by Chi-square analysis). The percentage of patients with multi-vessel disease was only 16.2% (44/271 patients) in the VHD (+) group but 22.0% (700/3,186 patients) in the VHD (-) group. In addition, the percentage of patients with CAD was only 5.9% (44/744 patients) in the multi-vessel disease (+) group and 8.4% (227/2,713 patients) in the multi-vessel disease (-) group. The percentage of patients with multi-vessel disease was 20.5% (27/132 patients) in AS, 13.9% (11/79 patients) in AR, 15.0% (3/20 patients) in MS, and only 8.0% (8/100 patients) in MR.

**Table 4:** Results of the Chi-square analysis of the study population with and without valvular heart disease or multi-vessel coronary disease.

*P =* 0.031 (N = 3,457)	Multi-vessel CAD (-) (n = 2,713)	Multi-vessel CAD (+) (n = 744)
VHD (-) (n = 3,186)	2,486	700
VHD (+) (n = 271)	227	44

Multi-vessels coronary disease means 2 or 3 vessel coronary artery disease.

CAD, Coronary artery disease and VHD, valvular heart disease

### Multivariate analysis to search the factors exerting an opposing effect on VHD and CAD

To search for a possible factor having an opposite effect on VHD and CAD among the conventional risk factors, we performed multiple logistic regression analysis. As shown in Table 5, age, male gender, HbA1c and dyslipidemia were positively associated with VHD and CAD, respectively. Hypertension was associated with CAD but not with VHD. In addition, smoking and obesity were not significant factors for VHD and CAD. As just described, we could not find factors exerting an opposing effect on VHD and CAD among the conventional risk factors.

**Table 5:** Results of multivariate analyses for the contribution of risk factors to valvular heart disease or coronary artery disease.

N = 3,457	Logistic regression analysis for VHD		Linear regression analysis for CAD	
	(n = 252, R^2^ = 0.120*)		(n = 628, R^2^ = 0.068)	
	*β* (Standardized coefficient) [95% CI]	*P* value	*β* (Standardized coefficient) [95% CI]	*P* value
Age	1.031 [1.017–1.044]	<0.001	0.120 [0.090, 0.161]	<0.001
Gender	0.499 [0.368–0.676]	<0.001	0.098 [0.057, 0.123]	<0.001
Hypertension	0.981 [0.707–1.360]	0.909	0.068 [0.039, 0.106]	<0.001
Current smoker	0.795 [0.536–1.181]	0.257	0.029 [0.002, 0.069]	0.108
HbA1c	0.588 [0.485–0.712]	<0.001	0.153 [0.116, 0.187]	<0.001
BMI	0.994 [0.959–1.030]	0.721	-0.015 [-0.043, 0.029]	0.455
Dyslipidemia	0.393 [0.299–0.516]	<0.001	0.077 [0.050, 0.116]	<0.001
Constant	1.090	0.911	-	-

HbA1c, hemoglobin A1c; BMI, body mass index.

CAD, Coronary artery disease; VHD, valvular heart disease; CI, confidence interval; and R^2^, squared multiple correlations (*: Nagelkerke)

### Concept of proposed path model C using the plasma BNP levels

Here, we proposed the possibility of plasma BNP, which was differently linked to VHD and CAD. Theoretical path model C was then proposed by positioning VHD as a cause of an increase in plasma BNP, and BNP and CAD were allocated as affecting each other. Paths between variables were drawn from independent to dependent variables with a directional arrow for every regression model, which enabled examining causality.

### Result of path model C

**Fig 4:**
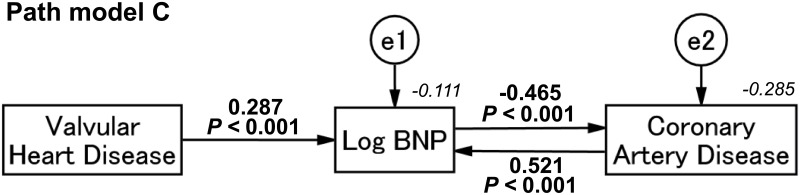
Path model (C): Explanatory drawing of the possible cascade among valvular heart disease, plasma BNP and coronary artery disease. This path has a coefficient showing the standardized coefficient of regressing independent variables on the dependent variable of the relevant path. These variables indicate standardized regression coefficients and squared multiple correlations [italic capitals]. BNP, B-type natriuretic peptide.

As shown in Fig 4 and Table 6, path model C revealed that VHD was causally associated with an increase in plasma BNP (standardized regression coefficients, [*β*]: 0.287, 95% confidence interval [CI], [0.251, 0.330], *P* < 0.001). Moreover, an increase in plasma BNP then suppressed the severity of CAD (*β*: -0.465, 95% CI, [-0.682, -0.334], *P* < 0.001). In reverse, this analysis showed that CAD augmented the activity of BNP (*β*: 0.521, 95% CI, [0.401, 0.720], *P* < 0.001). We identified an intrinsic cascade from the presence of VHD to the suppression of CAD through the activation of BNP.

**Table 6:** Results of path model C.

Clinical factor			Regression coefficient [95% CI]	*P* value	Standardized regression coefficient [95% CI]		
					Direct effects	Indirect effects	Total effects
Log BNP (R^2^ = -0.111)	←	VHD	0.697 [0.609, 0.803]	<0.001	0.287 [0.251, 0.330]	-0.056 [-0.103, -0.032]	0.231 [0.193, 0.260]
	←	CAD	0.377 [0.291, 0.520]	<0.001	0.521 [0.401, 0.720]	-0.102 [-0.237, -0.048]	0.420 [0.353, 0.483]
	←	Log BNP	-		-	-0.195 [-0.330, -0.118]	-0.195 [-0.330, -0118]
CAD (R^2^ = -0.285)	←	Log BNP	-0.641 [-0.949, -0.459]	<0.001	-0.465 [-0.682, -0.334]	0.091 [0.040, 0.225]	-0.374 [-0.458, -0.294]
	←	VHD	-		-	-0.107 [-0.145, -0.079]	-0.107 [-0.145, -0.079]
	←	CAD	-		-	-0.195 [-0.330, -0.118]	-0.195 [-0.330, -0.118]

The direct effects of the path model theoretically proposed (see Fig 4).

CAD, Coronary artery disease; VHD, valvular heart disease; e, extraneous variable; CI, confidence interval; and R^2^, squared multiple correlations

## Discussion

At the beginning of this study, we identified the expression frequency of VHD and CAD in consideration of aging (and/or age-associated factors) as a recognizable style (Fig 1). Each disorder was seemingly related to aging (and/or age-associated factors), and it is thus natural to consider the possible presence of comorbid conditions between the two disorders. However, the true relationship was unclear. To perform the statistical analysis, we used covariance structure analysis and proposed a hierarchical equation model. The path model could be constructed as a model akin to the image in Fig 1.

Consequently, the path model showed no significant positive association between VHD and CAD; rather, their association was a conflicting relationship, which became a matter of deep interest. The appearance of the comorbid condition was negative by this analysis. Although the value of the correlation coefficient (e1-e2, β: -0.121, *P* < 0.001) generally appears not to be high, we believe that the inverse relationship is strong because the negative impact between them (the conflicting relationship) produced the inversion phenomenon despite the existence of a common cascade, an age-related atherosclerosis process in VHD and CAD.

In the second study, to closely examine the causes of the inverse association between VHD and CAD, we performed a multivariate analysis using the conventional risk factors as shown in Table 5. Some factors contributed to VHD and some to CAD. However, no factors were inversely associated with VHD and CAD. These factors do not explain the conflicting relationship. In this view, some different pathologic mechanisms may contribute to the formation of VHD and CAD, even though the atherosclerotic and calcific process based on aging are involved in the common pathogenesis of VHD and CAD.

As stated above, the presence of an intervention factor that suppresses one or the other ought to be considered. One of the possible interjacent factors may be the activation of natriuretic peptide.

During the VHD formation process over years or decades, systemic arteries, including the coronary arteries, are exposed to high levels of circulating natriuretic peptide; therefore, the arterial predisponency would differ between the conditions of high and low natriuretic peptide levels. In an extreme case, VHD with high-plasma BNP levels may favor slowing the atherosclerotic process of CAD. On the other hand, as we recently reported, the plasma BNP levels were relatively low in patients with CAD compared with those with non-CAD [17]. Theoretically, the difference in the activity of natriuretic peptide should yield a difference in the pathologic process and pace of CAD development. Fig 4 and Table 6 are the results of a path model we proposed to this end. This analysis would strongly support our hypothesis because the standardized coefficient of regression was substantially strong (from VHD to Log BNP: 0.287; from Log BNP to CAD: -0.465).

In general, myocardial infarction or severe ischemia is sometimes responsible for MR. CAD can thus damage the area of the cardiac myocytes that supports the mitral valve, affecting valve function. In this regard, CAD and MR may occur simultaneously. However, even allowing for this event, there was only 8.0% multi-vessel disease in MR, as shown in Table 4, while 22.0% was found in VHD (-). This also supports a lower frequency of MR in CAD.

As shown in path model B, every subtype of VHD—AS, AR, MS, and MR—showed a significant and inverse association with CAD. Among the subtypes of VHD, AS should become an important disease in the future because, since the progression of AS is associated with female gender and aging, the incidence of AS will increase along with the aging female population. Thus, the contribution of atherosclerosis and calcific process to AS formation grows progressively by aging, which becomes synonymous with atherosclerosis and calcific process akin to that in CAD. Thus, AS will be an independent predictive marker for CAD in patients hospitalized for chest pain, and it should be considered in the risk stratification of these patients [34].

### Study limitations

First, the current study showed an inverse relationship between VHD and CAD. However, the value of the correlation coefficient (e1-e2, β: -0.121) still generally appears not to be high. A similar analysis should be performed in much larger population to confirm the conflicting relationship. Second, we speculated that natriuretic peptide is an interjacent factor between VHD and CAD; however, other significant factors likely exist and should be investigated.

## Conclusion

The incidence of VHD and CAD showed a significant conflicting relationship. This result supported the likely presence of unknown diverse mechanisms in addition to the common cascade of atherosclerosis and calcification. Among them, the continuous elevation of plasma BNP due to VHD might be an explicable factor.

## References

[1] Cilliers AM. Rheumatic fever and its management. BMJ. 2006 12 2;333(7579):1153–6. Review. doi: 10.1136/bmj.39031.420637.BE . 17138996 10.1136/bmj.39031.420637.BE PMC1676147

[2] Freeman RV, Otto CM. Spectrum of calcific aortic valve disease: pathogenesis, disease progression, and treatment strategies. Circulation. 2005 6 21;111(24):3316–26. doi: 10.1161/CIRCULATIONAHA.104.486738 . 15967862 10.1161/CIRCULATIONAHA.104.486738

[3] Korn D, Desanctis RW, Sell S. Massive calcification of the mitral annulus. A clinicopathological study of fourteen cases. N Engl J Med. 1962 11 1;267:900–9. doi: 10.1056/NEJM196211012671802 . 14034804 10.1056/NEJM196211012671802

[4] Roberts WC. Morphologic features of the normal and abnormal mitral valve. Am J Cardiol. 1983 3 15;51(6):1005–28. doi: 10.1016/S0002-9149(83)80181-7 . 6338691 10.1016/s0002-9149(83)80181-7

[5] Adler Y, Fink N, Spector D, Wiser I, Sagie A. Mitral annulus calcification—a window to diffuse atherosclerosis of the vascular system. Atherosclerosis. 2001 3;155(1):1–8. doi: 10.1016/S0021-9150(00)00737-1 . 11223420 10.1016/s0021-9150(00)00737-1

[6] Allison MA, Cheung P, Criqui MH, Langer RD, Wright CM. Mitral and aortic annular calcification are highly associated with systemic calcified atherosclerosis. Circulation. 2006 2 14;113(6):861–6. Epub 2006 Feb 6. doi: 10.1161/CIRCULATIONAHA.105.552844 . 16461818 10.1161/CIRCULATIONAHA.105.552844

[7] Pressman GS, Crudu V, Parameswaran-Chandrika A, Romero-Corral A, Purushottam B, Figueredo VM. Can total cardiac calcium predict the coronary calcium score? Int J Cardiol. 2011 1 21;146(2):202–6. doi: 10.1016/j.ijcard.2009.06.057 Epub 2009 Jul 17. . 19615766 10.1016/j.ijcard.2009.06.057

[8] Holtz JE, Upadhyaya DS, Cohen BE, Na B, Schiller NB, Whooley MA. Mitral annular calcium, inducible myocardial ischemia, and cardiovascular events in outpatients with coronary heart disease (from the Heart and Soul Study). Am J Cardiol. 2012 4 15;109(8):1092–6. doi: 10.1016/j.amjcard.2011.11.043 Epub 2012 Jan 14. . 22245404 10.1016/j.amjcard.2011.11.043

[9] Brasch E, Gottdiener JS, Larsen EK, Chaves PH, Newman AB, Manolio TA. Clinical significance of calcification of the fibrous skeleton of the heart and aortosclerosis in community dwelling elderly. The Cardiovascular Health Study (CHS). Am Heart J. 2006 1;151(1):39–47. doi: 10.1016/j.ahj.2005.03.052 . 16368289 10.1016/j.ahj.2005.03.052

[10] Mukoyama M, Nakao K, Hosoda K, Suga S, Saito Y, Ogawa Y, et al Brain natriuretic peptide as a novel cardiac hormone in humans. Evidence for an exquisite dual natriuretic peptide system, atrial natriuretic peptide and brain natriuretic peptide J Clin Invest. 1991;87:1402–1412. doi: 10.1172/JCI115146 . 1849149 10.1172/JCI115146 PMC295184

[11] Yasue H, Yoshimura M, Sumida H, Kikuta K, Kugiyama K, Jougasaki M, et al Localization and mechanism of secretion of B-type natriuretic peptide in comparison with those of A-type natriuretic peptide in normal subjects and patients with heart failure. Circulation. 1994 7;90(1):195–203. doi: 10.1161/01.CIR.90.1.195 . 8025996 10.1161/01.cir.90.1.195

[12] Yoshimura M, Yasue H, Okumura K, Ogawa H, Jougasaki M, Mukoyama M, et al Different secretion patterns of atrial natriuretic peptide and brain natriuretic peptide in patients with congestive heart failure. Circulation. 1993 2;87(2):464–9. doi: 10.1161/01.CIR.87.2.464 . 8425293 10.1161/01.cir.87.2.464

[13] Omland T. B-type natriuretic peptides: prognostic markers in stable coronary artery disease. Expert Rev Mol Diagn. 2008 3;8(2):217–25. doi: 10.1586/14737159.8.2.217 . 18366308 10.1586/14737159.8.2.217

[14] Oremus M, Raina PS, Santaguida P, Balion CM, McQueen MJ, McKelvie R, et al A systematic review of BNP as a predictor of prognosis in persons with coronary artery disease. Clin Biochem. 2008 3;41(4–5):260–5. Epub 2007 Sep 25. doi: 10.1016/j.clinbiochem.2007.09.011 . 17949703 10.1016/j.clinbiochem.2007.09.011

[15] Clerico A, Giannoni A, Vittorini S, Emdin M. The paradox of low BNP levels in obesity. Heart Fail Rev. 2012 1;17(1):81–96. doi: 10.1007/s10741-011-9249-z . 21523383 10.1007/s10741-011-9249-z

[16] Kawai M, Yoshimura M, Harada M, Mizuno Y, Hiramitsu S, Shimizu M, Shoda T, Kuwahara K, Miyagishima K, Ueshima K, Nakao K. Determination of the B-type natriuretic peptide level as a criterion for abnormalities in Japanese individuals in routine clinical practice: the J-ABS Multi-Center Study (Japan Abnormal BNP Standard). Intern Med. 2013;52(2):171–7. Epub 2013 Jan 15. . 23318845 10.2169/internalmedicine.52.8704

[17] Minai K, Ogawa T, Kawai M, Komukai K, Tanaka T, Ogawa K, et al The plasma B-type natriuretic peptide levels are low in males with stable ischemic heart disease (IHD) compared to those observed in patients with non-IHD: a retrospective study. PLoS One. 2014 10 31;9(10):e108983 doi: 10.1371/journal.pone.0108983 eCollection 2014. . 25360594 10.1371/journal.pone.0108983 PMC4215845

[18] Inoue Y, Kawai M, Minai K, Ogawa K, Nagoshi T, Ogawa T, et al The impact of an inverse correlation between plasma B-type natriuretic peptide levels and insulin resistance on the diabetic condition in patients with heart failure. Metabolism. 2016 3;65(3):38–47. doi: 10.1016/j.metabol.2015.09.019 Epub 2015 Sep 25. . 26892514 10.1016/j.metabol.2015.09.019

[19] Kinoshita K, Kawai M, Minai K, Ogawa K, Inoue Y, Yoshimura M. Potent influence of obesity on suppression of plasma B-type natriuretic peptide levels in patients with acute heart failure: An approach using covariance structure analysis. Int J Cardiol. 2016 7 15;215:283–90. doi: 10.1016/j.ijcard.2016.04.111 Epub 2016 Apr 16. . 27128547 10.1016/j.ijcard.2016.04.111

[20] Yoshida J, Kawai M, Minai K, Ogawa K, Ogawa T, Yoshimura M. Associations between left ventricular cavity size and cardiac function and overload determined by natriuretic peptide levels and a covariance structure analysis. Sci Rep. 2017 5 17;7(1):2037 doi: 10.1038/s41598-017-02247-5 . 28515459 10.1038/s41598-017-02247-5 PMC5435711

[21] Tsutsumi J, Minai K, Kawai M, Ogawa K, Inoue Y, Morimoto S, et al Manifold implications of obesity in ischemic heart disease among Japanese patients according to covariance structure analysis: Low reactivity of B-type natriuretic peptide as an intervening risk factor. PLoS One. 2017 5 8;12(5):e0177327 doi: 10.1371/journal.pone.0177327 eCollection 2017. . 28481950 10.1371/journal.pone.0177327 PMC5421780

[22] Miyashita K, Itoh H, Tsujimoto H, Tamura N, Fukunaga Y, Sone M, et al Natriuretic peptides/cGMP/cGMP-dependent protein kinase cascades promote muscle mitochondrial biogenesis and prevent obesity. Diabetes. 2009 12;58(12):2880–92. Epub 2009 Aug 18. doi: 10.2337/db09-0393 . 19690065 10.2337/db09-0393 PMC2780866

[23] Yamahara K, Itoh H, Chun TH, Ogawa Y, Yamashita J, Sawada N, et al Significance and therapeutic potential of the natriuretic peptides/cGMP/cGMP-dependent protein kinase pathway in vascular regeneration. Proc Natl Acad Sci U S A. 2003 3 18;100(6):3404–9. Epub 2003 Mar 5. doi: 10.1073/pnas.0538059100 . 12621153 10.1073/pnas.0538059100 PMC152305

[24] Nishimura RA, Otto CM, Bonow RO, Carabello BA, Erwin JP 3rd, Fleisher LA, et al 2017 AHA/ACC Focused Update of the 2014 AHA/ACC Guideline for the Management of Patients With Valvular Heart Disease: A Report of the American College of Cardiology/American Heart Association Task Force on Clinical Practice Guidelines. Circulation. 2017 3 15 doi: 10.1161/CIR.0000000000000503 . 28298458 10.1161/CIR.0000000000000503

[25] Zoghbi WA, Enriquez-Sarano M, Foster E, Grayburn PA, Kraft CD, Levine RA, et al; American Society of Echocardiography. Recommendations for evaluation of the severity of native valvular regurgitation with two-dimensional and Doppler echocardiography. J Am Soc Echocardiogr. 2003 7;16(7):777–802. doi: 10.1016/S0894-7317(03)00335-3 . 12835667 10.1016/S0894-7317(03)00335-3

[26] Komukai K, Ogawa T, Yagi H, Date T, Sakamoto H, Kanzaki Y, et al Decreased renal function as an independent predictor of re-hospitalization for congestive heart failure. Circ J. 2008 7;72(7):1152–7. doi: 10.1253/circj.72.1152 . 18577827 10.1253/circj.72.1152

[27] Levey AS, Bosch JP, Lewis JB, Greene T, Rogers N, Roth D. A more accurate method to estimate glomerular filtration rate from serum creatinine: a new prediction equation. Modification of Diet in Renal Disease Study Group. Ann Intern Med. 1999 3 16;130(6):461–70. doi: 10.7326/0003-4819-130-6-199903160-00002 . 10075613 10.7326/0003-4819-130-6-199903160-00002

[28] Matsuo S, Imai E, Horio M, Yasuda Y, Tomita K, Nitta K, et al; Collaborators developing the Japanese equation for estimated GFR. Revised equations for estimated GFR from serum creatinine in Japan. Am J Kidney Dis. 2009 6;53(6):982–92. doi: 10.1053/j.ajkd.2008.12.034 Epub 2009 Apr 1. . 19339088 10.1053/j.ajkd.2008.12.034

[29] Clerico A, Zaninotto M, Prontera C, Giovannini S, Ndreu R, Franzini M, et al; Study Group on Cardiovascular Risk Biomarkers of the Italian Society of Clinical Biochemistry. State of the art of BNP and NT-proBNP immunoassays: the CardioOrmoCheck study. Clin Chim Acta. 2012 12 24;414:112–9. doi: 10.1016/j.cca.2012.07.017 Epub 2012 Aug 15. . 22910582 10.1016/j.cca.2012.07.017

[30] Ogawa K, Minai K, Kawai M, Tanaka T, Nagoshi T, Ogawa T, et al Parallel comparison of risk factors between progression of organic stenosis in the coronary arteries and onset of acute coronary syndrome by covariance structure analysis. PLoS One. 2017 3 16;12(3):e0173898 doi: 10.1371/journal.pone.0173898 eCollection 2017. . 28301565 10.1371/journal.pone.0173898 PMC5354387

[31] Ito S, Nagoshi T, Minai K, Kashiwagi Y, Sekiyama H, Yoshii A, et al Possible increase in insulin resistance and concealed glucose-coupled potassium-lowering mechanisms during acute coronary syndrome documented by covariance structure analysis. PLoS One. 2017 4 21;12(4):e0176435 doi: 10.1371/journal.pone.0176435 eCollection 2017. . 28430816 10.1371/journal.pone.0176435 PMC5400267

[32] Tanaka Y, Nagoshi T, Kawai M, Uno G, Ito S, Yoshii A, et al Close linkage between serum uric acid and cardiac dysfunction in patients with ischemic heart disease according to covariance structure analysis. Sci Rep. 2017 5 30;7(1):2519 doi: 10.1038/s41598-017-02707-y . 28559584 10.1038/s41598-017-02707-y PMC5449391

[33] Shumaker RE & Lomax RG A beginner’s guide to structural equation modeling. Furth edition New York: Published by Routledge (on imprint of the Taylor & Francis Group); 2016.

[34] Conte L, Rossi A, Cicoira M, Bonapace S, Amado EA, Golia G, et al Aortic valve sclerosis: a marker of significant obstructive coronary artery disease in patients with chest pain? J Am Soc Echocardiogr. 2007 6;20(6):703–8. doi: 10.1016/j.echo.2006.11.018 . 17543740 10.1016/j.echo.2006.11.018

